# Supplementary material for: Improved resolution of avian influenza virus using Oxford Nanopore R10 sequencing chemistry
Source: Microbiol Spectr. 2024 Nov 7;12(12):e01880-24. doi: 10.1128/spectrum.01880-24 (PMC11623064; doi:10.1128/spectrum.01880-24)
Supplement: Supplemental material — Supplemental methods and figures. [file spectrum.01880-24-s0001.pdf]

Supplementary Methods

Sequence composition

The potential impact of base composition was assessed by grouping 9mers according to their relative indel rate or their favored chemistry. Top 10% cutoff values were determined for insertion and deletion rates for both chemistries using the quantile() function in the *stats* package in R. To assess composition, 9mers were concatenated and individual bases of "A", "T", "C", or "G" were summed using the str_count function from the *stringr* package. Global differences in composition were tested with a Chi-squared test; significant associations were further interrogated using a two-sample proportion test. Homopolymer lengths of 3+, 4+, or 5+ were detected using the base *grep* function in R. Differences in proportions of 9mers with homopolymers were tested using a Chi-squared test.

Supplementary Results

9mer composition influences observed deletion rate in R9.4.1 and R10.4.1 flow cells

Previous studies have reported an influence of GC content on nanopore error rates [1]. To investigate any role in base composition in observed indel rates in this study, the 29,705 analyzed 9mers were grouped into those with the top 10% highest deletion rates and those in the bottom 90% for each chemistry. This segregation was repeated for insertions. Results are summarized in table S2.

For deletions, composition of the top 10% compared to the bottom 90% was significantly different for both R9.4.1 and R10.4.1 ($p = 0.0014$ and $p = 0.0037$, respectively). For R9.4.1, post-hoc tests suggest an overrepresentation of guanines ($p = 0.018$) and underrepresentation of cytosines ($p = 0.0015$) in the top 10%. For R10.4.1, guanines and thymines are significantly overrepresented ($p = 0.036$ and $p = 0.033$) while adenines are significantly underrepresented ($p = 0.0058$). Despite the different effects suggested by the post-hoc tests, the composition of the 9mers with the top 10% deletion rates in R9.4.1 were not significantly different than those in the top 10% deletion rates in R10.4 ($p = 0.52$, chi-squared test). Further, there were no significant differences in composition for any comparisons of insertion rate groupings.

The above results suggested a potential subtle difference in the influence of composition on the indel rate R9.4.1 and R10.4.1 flow cells. To further assess this hypothesis, the base composition of 9mers with significant differences in performance across the flow cells were compared. There was no difference for those 9mers with significantly favored chemistries for deletions ($p = 0.27$). Interestingly, the composition of those 9mers with significantly favored chemistries for insertions was significantly different ($p = 4.0e-5$). Post-hoc proportion tests revealed significant differences for adenines, cytosines, and guanines ($p = 0.000040$, 0.00065, and 0.038, respectively).

There were no associations observed in the frequency with which homopolymers of lengths 3+, 4+, or 5+ were present in the 9mers favored by either chemistry for insertions or deletions (Table S3).

Supplementary Tables and Figures

Table S1: List of Samples Sequenced for This Study

Isolate_Name	Host	Original or Passage	FluA qPCR(Ct)	PCR Subtype	Seq. Subtype	Isolate ID	Segment ID HA	Segment ID NA	MBCS	Phenotype*
A/Cambodia/g1004362/2022	human	siat, P1	10.63	H3N2	H3N2	17995509	EPI2630162	EPI2630161	Not present	
A/Cambodia/g1115365/2022	human	siat, P1	11.64	H3N2	H3N2	17995510	EPI2630170	EPI2630169	Not present	
A/chicken/Cambodia/f6PPOreu241C7T/2021	poultry	P1e1	17.96	H5N8	H5N8	18060556	EPI2666610	EPI2666609	REKRRKR↓GLF	HPAI
A/chicken/Cambodia/g14T241C4C/2022	poultry	P2e2	11.34	H5N8	H5N8	18060557	EPI2666612	EPI2666611	REKRRKR↓GLF	HPAI
A/duck/Cambodia/g14T241D14T/2022	poultry	P2e2	13.49	H5N8	H5N8	18060558	EPI2666614	EPI2666613	REKRRKR↓GLF	HPAI
A/duck/Cambodia/g14T241D8T/2022	poultry	P1e3	12.08	H5N8	H5N8	18060559	EPI2666616	EPI2666615	REKRRKR↓GLF	HPAI
A/duck/Cambodia/g15T241D6T/2022	poultry	P2e1	10.78	H5N8	H5N8	18060560	EPI2666618	EPI2666617	REKRRKR↓GLF	HPAI
A/duck/Cambodia/g14T241D12C/2022	poultry	P1e2	10.59	H5N8	H5N8	18060561	EPI2666620	EPI2666619	REKRRKR↓GLF	HPAI
A/duck/Cambodia/f7T241D10T/2021	poultry	P1e1	11.07	H5N2	H5N2	18060562	EPI2666622	EPI2666621	REKRRKR↓GLF	HPAI
A/duck/Cambodia/f7T241D3T/2021	poultry	P1e2	10.93	H5N2	H5N2	18060563	EPI2666624	EPI2666623	REKRRKR↓GLF	HPAI
A/duck/Cambodia/f7T241D12T/2021	poultry	P1e1	9.22	H5N2	H5N2	18060564	EPI2666626	EPI2666625	REKRRKR↓GLF	HPAI
A/duck/Cambodia/f7T241D17T/2021	poultry	P1e3	13.42	H5N2	H5N2	18060565	EPI2666628	EPI2666627	REKRRKR↓GLF	HPAI
A/duck/Cambodia/g9K241D3T/2022	poultry	p1e1	13.05	H5N1	H5N1	18060566	EPI2666630	EPI2666629	KERRRKR↓GLF	HPAI
A/duck/Cambodia/h4T241D5C/2023	poultry	original	24.01	H5N1	H5N1	18060567	EPI2666632	EPI2666631	RERRRKR↓GLF	HPAI
A/duck/Cambodia/h4T241D17C/2023	poultry	original	24.39	H5N1	H5N1	18060568	EPI2666634	EPI2666633	RERRRKR↓GLF	HPAI
A/duck/Cambodia/h4PPChba241D4T/2023	poultry	P1e3	9.78	H5N1	H5N1	18060569	EPI2666636	EPI2666635	KERRRKR↓GLF	HPAI
A/duck/Cambodia/h4PPChba241D5T/2023	poultry	P1e3	12.94	H5N1	H5N1	18060570	EPI2666638	EPI2666637	KERRRKR↓GLF	HPAI
A/duck/Cambodia/h4PPChba241D6T/2023	poultry	P1e3	14.56	H5N1	H5N1	18060571	EPI2666640	EPI2666639	KERRRKR↓GLF	HPAI
A/duck/Cambodia/h4PPChba241D7T/2023	poultry	P1e1	11.9	H5N1	H5N1	18060572	EPI2666642	EPI2666641	KERRRKR↓GLF	HPAI
A/duck/Cambodia/h4PPChba241D9T/2023	poultry	P1e2	11.29	H5N1	H5N1	18060573	EPI2666644	EPI2666643	KERRRKR↓GLF	HPAI
A/chicken/Cambodia/g1PPChba241C7T/2023	poultry	p1e1	12.93	H5N1	H5N1	18060574	EPI2666646	EPI2666645	KERRRKR↓GLF	HPAI
A/duck/Cambodia/g1PPOreu241D6T/2023	poultry	original	20.81	H5N1	H5N1	18060575	EPI2666648	EPI2666647	KERRRKR↓GLF	HPAI
A/chicken/Cambodia/g1PPChba241C9T/2023	poultry	original	19.18	H5N1	H5N1	18060576	EPI2666650	EPI2666649	KERRRKR↓GLF	HPAI
A/duck/Cambodia/h2PPChba241D2/2023	poultry	original	24.23	H5N1	H5N1	18949137	EPI3086766	EPI3086765	KERRRKR↓GLF	HPAI

A/chicken/Cambodia/h2T241C8T/2023	poultry	original	24.24	H9N2	H9N2	18949138	EPI3086768	EPI3086767	Not present	LPAI
A/duck/Cambodia/h3PPOreu241D9C/2023	poultry	original	21.27	FluA	H6N6	18949139	EPI3086770	EPI3086769	Not present	LPAI
A/duck/Cambodia/h3PPOreu241D10C/2023	poultry	original	22.35	FluA	H6N6	18949140	EPI3086772	EPI3086771	Not present	LPAI
A/duck/Cambodia/h3K241D6T/2023	poultry	original	24.16	H5Nx	H5N1	18949141	EPI3086774	EPI3086773	KERRRKR↓GLF	HPAI
A/duck/Cambodia/h3K241D7T/2023	poultry	original	23.26	H5N1	H5N1	18949142	EPI3086776	EPI3086775	KERRRKR↓GLF	HPAI
A/duck/Cambodia/h3K241D8T/2023	poultry	original	24.05	H5N1	H5N1	18949143	EPI3086778	EPI3086777	KERRRKR↓GLF	HPAI
A/chicken/Cambodia/h3T241C7T/2023	poultry	original	19.39	H5Nx	H5N1	18949144	EPI3086780	EPI3086779	KERRRKR↓GLF	HPAI
A/chicken/Cambodia/h3T241C9T/2023	poultry	original	20.12	H5Nx	H5N1	18949145	EPI3086782	EPI3086781	KERRRKR↓GLF	HPAI
A/chicken/Cambodia/h3T241C7C/2023	poultry	original	23.98	H5Nx	H5N1	18949146	EPI3086784	EPI3086783	KERRRKR↓GLF	HPAI
A/duck/Cambodia/h4T241D2T/2023	poultry	original	21.76	FluA	H5N1	18949147	EPI3086786	EPI3086785	RERRRKR↓GLF	HPAI
A/duck/Cambodia/h4T241D5T/2023	poultry	original	23.04	FluA	H5N1	18949148	EPI3086788	EPI3086787	RERRRKR↓GLF	HPAI
A/duck/Cambodia/h4T241D14T/2023	poultry	original	23.22	FluA	H5N1	18949149	EPI3086790	EPI3086789	RERRRKR↓GLF	HPAI
A/duck/Cambodia/h4T241D15T/2023	poultry	original	23.51	FluA	H5N1	18949150	EPI3086792	EPI3086791	RERRRKR↓GLF	HPAI
A/duck/Cambodia/h4T241D19T/2023	poultry	original	22.36	FluA	H5N1	18949151	EPI3086794	EPI3086793	RERRRKR↓GLF	HPAI
A/duck/Cambodia/h4T241D20T/2023	poultry	original	22.07	FluA	H5N1	18949152	EPI3086796	EPI3086795	RERRRKR↓GLF	HPAI
A/environment/Cambodia/h2T241WW1E/2023	environ ment	original	23.53	H5Nx	H5N1	18949153	EPI3086798	EPI3086797	KERRRKR↓GLF	HPAI
A/environment/Cambodia/h2T241WW5E/2023	environ ment	original	22.99	H5N1/H9 N2	H5N1	N/A#	N/A	N/A	mixed	mixed
A/environment/Cambodia/h3T241WW1E/2023	environ ment	original	22.52	H5Nx/H9 Nx	H5N1	N/A#	N/A	N/A	mixed	mixed

*HPAI = highly pathogenic avian influenza; LPAI = low pathogenic avian influenza

#N/A = not available; sequence not submitted to GISAID because of mixed subtypes in sample

Table S2: Comparisons of base composition between 9mers of discordant indel rates. Comparisons significant with an alpha value of 0.05 are highlighted in light grey; comparisons significant after a Bonferroni correction for 20 comparisons (alpha = 0.0025) are highlighted in dark grey.

Indel	Group 1	Group 2	Composition comparison	Group 1 value (counts or %)	Group 2 value (counts or %)	P value	Statistical test
Deletion	R10.4.1, top 10% highest rates	R10.4.1, bottom 90% lowest rates	Overall	A: 8217<br>C: 6533<br>T: 5330<br>G: 6659	A: 75931<br>C: 57369<br>T: 48784<br>G: 58513	0.0037	Chi-squared test
			Adenine	30.7%	31.6%	0.0058	Two-sample proportion test
			Cytosine	19.9%	20.3%	0.19	
			Thymine	24.4%	23.8%	0.033	
			Guanine	24.9%	24.3%	0.036	
	R9.4.1, top 10% highest rates	R9.4.1, bottom 90% lowest rates	Overall	A: 8340<br>C: 6508<br>T: 5214<br>G: 6677	A: 75808<br>C: 57394<br>T: 48900<br>G: 58495	0.0014	Chi-squared test
			Adenine	31.2%	31.5%	0.29	Two-sample proportion test
			Cytosine	24.3%	23.9%	0.080	
			Thymine	19.5%	20.3%	0.0015	
			Guanine	25.0%	24.3%	0.018	
	R10.4.1, top 10% highest rates	R9.4.1, top 10% highest rates	Overall	A: 8217<br>C: 6533<br>T: 5330<br>G: 6659	A: 8340<br>C: 6508<br>T: 5214<br>G: 6677	0.52	Chi-squared test
	R10 favored 9mers	R9 favored 9mers	Overall	A: 23909<br>C: 18224<br>T: 15286<br>G: 18550	A: 2887<br>C: 2288<br>T: 1905<br>G: 2370	0.27	Chi-squared test
Insertion	R10.4.1, top 10% highest rates	R10.4.1, bottom 90% lowest rates	Overall	A: 8330<br>C: 5512<br>T: 6364<br>G: 6533	A: 75818<br>C: 48602<br>T: 57538<br>G: 58639	0.36	Chi-squared test
	R9.4.1, top 10% highest rates	R9.4.1, bottom 90% lowest rates	Overall	A: 8402<br>C: 5269<br>T: 6477	A: 75746<br>C: 48845<br>T: 57425	0.093	Chi-squared test

				G: 6591	G: 58581		
	R10.4.1, top 10% highest rates	R9.4.1, top 10% highest rates	Overall	A: 8330<br>C: 5512<br>T: 6364<br>G: 6533	A: 8402<br>C: 5269<br>T: 6477<br>G: 6591	0.071	Chi-squared test
	R10 favored 9mers	R9 favored 9mers	Overall	A: 24840<br>C: 15294<br>T: 18390<br>G: 18795	A: 8117<br>C: 5477<br>T: 6211<br>G: 6583	2.8e-5	Chi-squared test
			Adenine	32.1%	30.8%	4.0e-5	Two-sample proportion test
			Cytosine	19.8%	20.8%	0.00065	
			Thymine	23.8%	23.5%	0.42	
			Guanine	24.3%	24.9%	0.038	

Table S3: Associations between Homopolymer Length and 9mer Membership. Baseline values - the proportion of 9mers within that category - reflect the null expectation of no association between homopolymer presence and favored chemistries. "Homopolymer lengths >= X" describe the proportion of 9mers that meet the homopolymer length criteria that are members of each category. No comparisons between baseline and "Homopolymer lengths >= X" were significant by Chi-Squared test.

Indel	Category	Baseline	Homopolymer length >= 3	Homopolymer length >= 4	Homopolymer length >= 5
Deletion	R10 favored	28.4% (8441/29705)	28.1% (3010/10722)	27.3% (727/2667)	28.5% (138/484)
	R9 favored	3.5% (1050/29705)	3.6% (388/10722)	3.5% (94/2667)	3.9% (19/484)
	None favored	68.0% (20214/29705)	68.3% (7324/10722)	69.2% (1846/2667)	67.6% (327/484)
Insertion	R10 favored	28.9% (8591/29705)	29.5% (3168/10722)	28.5% (760/2667)	28.5% (138/484)
	R9 favored	9.8% (2932/29705)	9.8% (1053/10722)	9.5% (253/2667)	9.3% (45/484)
	None favored	61.2% (18182/29705)	60.6% (6501/10722)	62.0% (1654/2667)	62.2% (301/484)

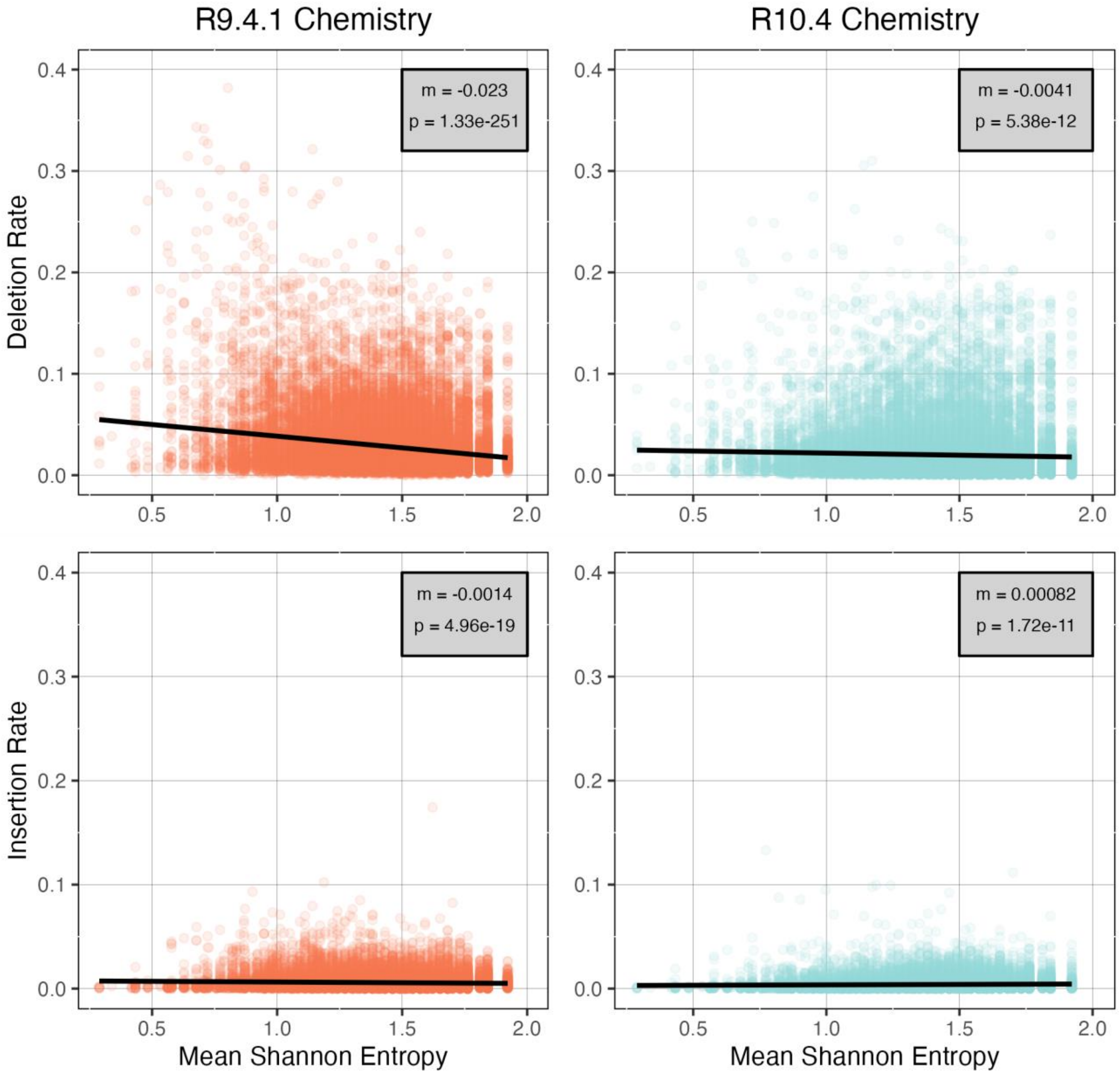

Figure S1: Correlations of mean shannon entropy values and observed indel rates. Dots represent individual 9mers (n = 29,705). M and p values derived from linear regression of the formula y = mx+b.

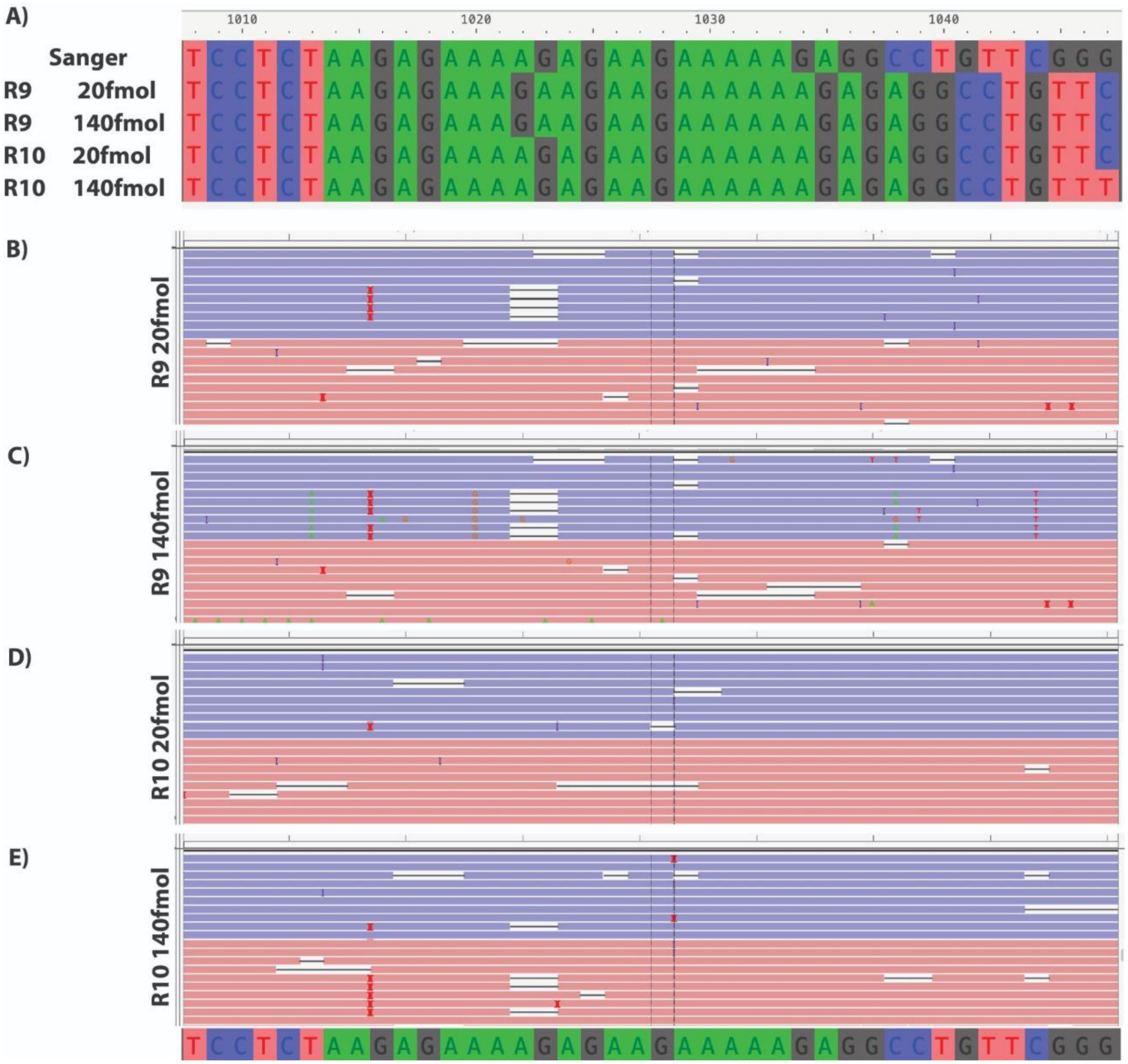

Figure S2: Reconciling consensus sequence differences between Sanger and Oxford Nanopore platforms. Consensus sequences generated from IRMA from all chemistries were aligned to a Sanger reference (A) for A/duck/Cambodia/g14T241D12C/2022 that produced conflicting results within the HA MBCS. Incorrect consensus insertions are outlined in red boxes. Following realignment of raw data to the Sanger reference (B-E), two types of insertions were observed. In R9 data, a deletion on the negative strand only resulted in an AG inversion (leftmost red box, B-C). In all data for this sample, issues resolving the adenine 5-mer were expanded by a full codon resulting in a repeated AGA motif at the end of the low-complexity region (rightmost red box, B-E).

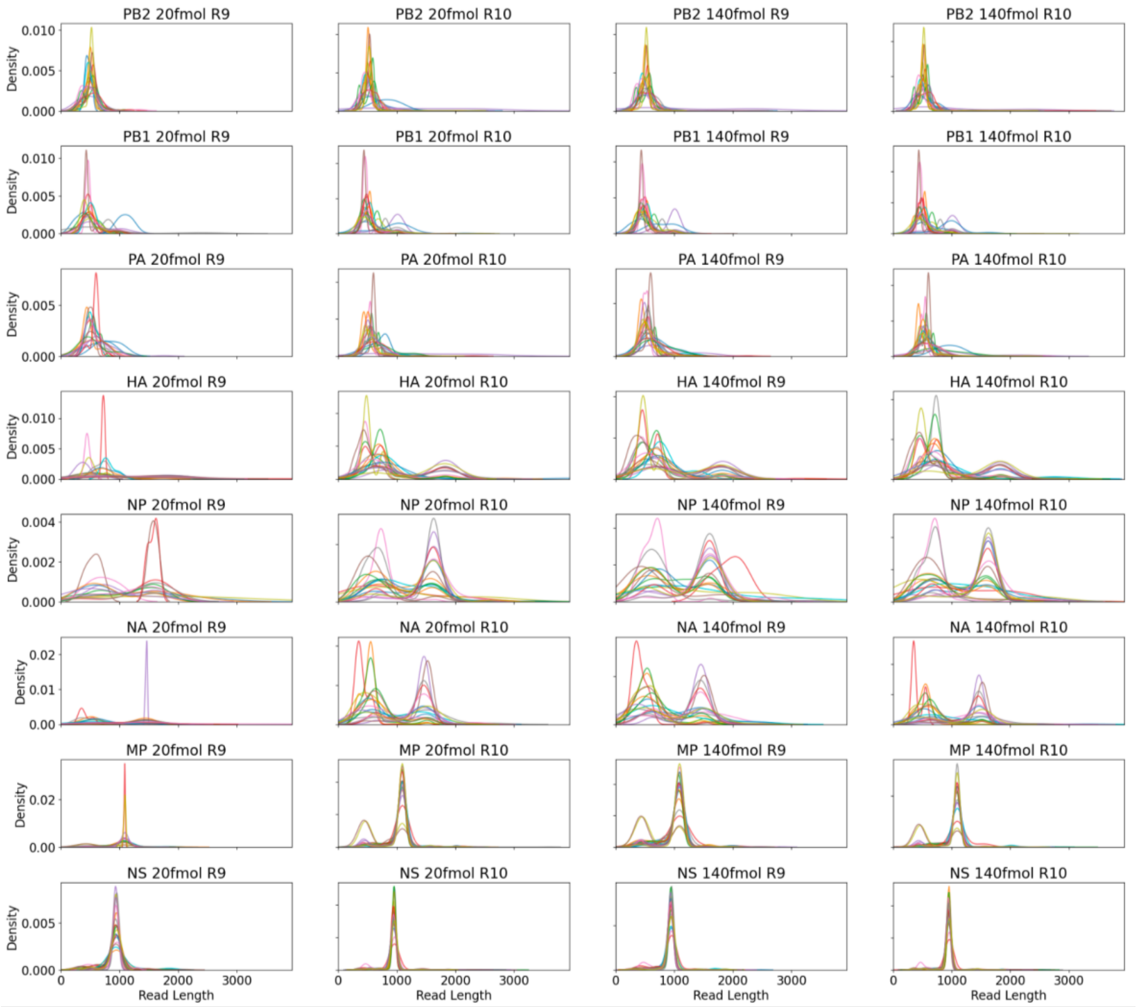

Figure S3: Distribution of coverage by sample and concentration per segment. Coverage was determined using samtools depth (1.16) to identify a kernel density estimation ridgeline plot per combination of sample, segment, and load concentration. A far greater aligned mean read length is observed with the NS and MP segments. Additionally, NA, NP, and HA see variability in the density of aligned readlengths.

Supplemental References:

1. Delahaye, C. & Nicolas, J. Sequencing DNA with nanopores: Troubles and biases. *PLoS One* **16**, e0257521 (2021).
